# Percutaneous navigable intradiscal decompression in treatment of lumbar disc herniation: a single-center experience

**DOI:** 10.3906/sag-1805-187

**Published:** 2019-04-18

**Authors:** Ayşegül CEYLAN, İbrahim AŞIK

**Affiliations:** 1 Department of Anesthesiology and Reanimation, Gülhane Training and Research Hospital,University of Medical Sciences, Ankara Turkey; 2 Department of Anesthesiology and Reanimation, Ankara University Faculty of Medicine Hospital, Ankara Turkey

**Keywords:** Navigable ablation, L-Disq, intradiscal decompression

## Abstract

**Background/aim:**

Minimally invasive procedures have been increasingly used for the treatment of herniated discs. Nonsurgical interventions minimize the secondary damage to other tissues and shorten the length of hospital stay by avoiding general anesthesia. Possible complications are thermal injuries, root injury, discitis, endplate damage, dural injury, meningitis, infection, increase in pain, and muscle spasm. We aimed to evaluate the efficacy of percutaneous decompression therapy by using intradiscal navigable electrodes on pain and functional movement index in patients with herniated nucleus pulposus (HNP).

**Materials and methods:**

A total of 209 patients with protrusive lumbar disc herniation underwent percutaneous ablation decompression treatment using an intradiscal routable electrode (L-Disq) in our pain clinic. Visual analog scale (VAS) and Oswestry Disability Index (ODI) scores were recorded at the beginning and at the 1st, 3rd, 6th, and 12th months after treatment. Patient satisfaction was evaluated at the 12th month by a patient satisfaction scale (PSS).

**Results:**

When compared to initial values, VAS and ODI scores showed statistically significant improvement at the 1st, 3rd, 6th, and 12th months (P < 0.001). Mean VAS scores were 7.28 and 3.03 points (P < 0.001) while mean ODI scores were 32.46 and 20.48 points (P < 0.001) at the beginning and at the 12th month, respectively. Satisfaction rate of all patients was 81%. We also attempted to treat the existing annular fissure using an ablation method and we believe that treating the herniated disc together with the fissure in the same session increased our success rate.

**Conclusion:**

With clinical evidence, we suggest that L-Disq may be considered as an appropriate option with a low risk of complications in pain management in cases of lumbar disc herniation that are resistant to conservative methods.

## 1. Introduction

Lumbar radiculopathy due to herniated nucleus pulposus (HNP) is associated with severe morbidity (1). A lumbar HNP compresses the nerve roots and mechanical and inflammatory mechanisms cause pain (2). The outcome after a microdiscectomy is worse in patients with small hernias than in those with sequestrated hernias (3), and this has led to a rise in the popularity of minimally invasive procedures for the treatment of herniated discs. Recently, with the increasing knowledge of spinal anatomy and the evidence that conventional procedures have not always been useful, there has been a tendency to carry out minimally invasive procedures for the treatment of disc herniation (4), which can be advantageous in terms of early recovery, early return to daily life, short operational times, relatively fewer surgical traumas, and less pain. Percutaneous disc decompression (PDD) methods are based on the principle that the removal of small amounts of discoid tissue will result in significant pain relief by reducing intradiscal pressure (5), as pressure on the nerve will be decreased and radicular findings will be reduced. There is also evidence that PDD can lead to a reduction in patients’ disability and increased safety (6–8).

Possible complications are thermal injuries, root injury, discitis, endplate damage, dural injury, meningitis, infection, increase in pain, and muscle spasm (9,10). All patients are given antibiotics for 7 days for prophylaxis of infection. So as to minimize direct nerve root irritation or damage during the procedure, our patients are kept awake and conscious, being in communication with the surgeon. Dural ruptures may sometimes occur and the treatment is hydration, rest, and medication. An epidural blood patching was planned for patients with remaining complaints of dural rupture. 

In this study, we evaluate the efficiency of PDD therapy using an intradiscal navigable electrode (L-Disq) on the pain and functional movement index in patients with HNP.

## 2. Materials and methods

After the approval of the ethics committee, clinical data from 209 patients with back/leg pain due to HNP who underwent ablation decompression treatment using L-Disq in our pain clinic between January 2013 and January 2017 were reviewed retrospectively.

### 2.1. Patient selection

Patients with disc herniation with a sagittal diameter greater than 33% of the spinal canal, narrowing of the discal space greater than 50%, previous lumbar spine surgery, sequestrated disc, vertebral fracture, spinal canal stenosis or spondylolisthesis, psychological problems detected during the examination, systemic or localized infection, history of tumor or coagulopathy, pregnancy, osteoarthritis, or a body mass index of ≥35 kg/m2 were excluded from the study. 

Patients were included if they were aged >18 years old and in the ASA I–II risk group; had no response to conservative treatments such as muscle relaxants, antiinflammatory agents, facet joint blockade, or epidural steroid injections for at least 3 months; and had waist and/or hip pain or secondary pain in the lower extremities associated with disc herniation. 

### 2.2. Material selection

For the analysis, we used an L-Disq navigable percutaneous decompression device (U&I Co. Ltd., Uijeongbu, Korea) that can reach the outer ring of a herniated and/or degenerated disc (11). 

### 2.3. Ablation decompression and other auxiliary procedures

All the decompression procedures were performed by a single experienced practitioner. The patients’ blood pressure, heart rate, electrocardiogram, oxygen saturation, and respiratory rate were monitored, and an aseptic technique was used throughout the whole procedure. Prior to the procedure, all patients were given 1 g of cefazolin for prophylaxis and 2 mg of midazolam to minimize anxiety and discomfort. In the case of pain, 0.5 µg/kg fentanyl was planned to be injected intravenously. The patients were calm, but alert and conscious, and could inform the practitioner in the event of unusual pain.

The patients were placed on the operating table in prone position and fluoroscopic images of the lumbar spine were obtained to confirm and determine the intervertebral disc levels and the appropriate level for needle entry. We used a standard posterolateral approach (12) to reach the L1–L4 intervertebral disc segments. The lumbar intervertebral level was marked 8–10 cm laterally from the midline on the interventional side under fluoroscopy. In the oblique position, we rotated the C-arm 90° from the front-rear position to a 30–35° lateral and 15° cephalic direction and then inserted the needle into the skin at a 30° angle. The most important procedural challenge during the intradiscal entry at the L5–S1 level was to overcome the positional and anatomical difficulty. Entry to the L5–S1 level at this angle is very difficult, and is even impossible in some patients. For this reason, while entering the L5–S1 segments, we inserted the needle 14–15 cm away from the vertebrae at an angle of 45° and by angling the scope 30° in the cephalic direction. The intervention through the L5–S1 level by changing the angle and site of the needle insertion and the scope angle greatly reduced the difficulties encountered due to anatomic location and contributed to the success of the procedure.

After injecting a local anesthetic agent, 2–3 mL (20 mg/mL) of prilocaine, into the cutaneous and subcutaneous tissues, the C-arm was placed to obtain a lateral view of the surgical field and an 18-gauge spinal needle of 8.9 cm was entered and pushed forward into the middle of the disc under fluoroscopy. Both anteroposterior (AP) and lateral views of the L-Disq electrode were obtained and the position within the disc was checked. The safety of the procedure was confirmed through negative motor nerve stimulations with short bursts in order to test the proximity of the electrode to the nerve root within the disc. Close monitoring of pain is necessary to prevent injuries from heat. In addition, if the electrical activity causes lower extremity stimulation, the rod must be straightened and moved into an open position. In all procedures, the bar was repeatedly rotated and moved back and forth in order to increase the ablated volume. 

If findings of disc degeneration (annular fissure) with disc protrusion were detected on MRI images, the fissure line was ablated by placing the tip of the electrode. The procedures lasted between 20 and 30 min.

If needed, patients with pain were given an antiinflammatory drug or paracetamol during the follow-up period. All patients were prescribed antibiotics to use for 7 days and instructed to take an antiinflammatory drug or paracetamol in case of pain. No patient required additional pain intervention after the procedure. 

After the procedure, patients were informed not to drive for at least 48 h, to start limited walking for 10–20 min after a few days, to obey the lifting limit of 5–10 kg for the first 2 weeks, not to bend or twist their waists, to avoid compulsive movements, to perform chiropractic manipulation for the first 12 weeks, to avoid massage or traction, to perform gentle flexion and extension movements at home for the first 2–3 weeks, and to perform specific physiotherapy for the first 3–5 weeks. 

If the patient had no contraindication, MRI images were taken at routine controls after the procedure. MRI findings were evaluated by the radiology clinic but the rate of change was not determined. All patients were evaluated using the Oswestry Disability Index (ODI) and a visual analog scale (VAS) according to the clinical data and the patient’s answers before and after treatment at the 1st, 3rd, 6th, and 12th months, and using a patient satisfaction scale (PSS) at the 12th month following treatment.

### 2.4. Statistical analysis

Statistical analyses were carried out using the SPSS 15 for Windows (SPSS Inc., Chicago, IL, USA). Descriptive statistics were expressed as mean ± standard deviation for variables with normal distribution, as median (min–max) for variables with abnormal distribution, and as number of cases and percentage (%) for nominal variables. Within the groups, the significance of the differences in median values and median values between times were assessed by the Friedman test. If present, multiple intertime comparisons of the differences were evaluated using appropriate post hoc tests.

Between the groups, the significance of the difference in terms of average values was evaluated by a two-related-samples test, and in terms of median values by the Wilcoxon test. P < 0.05 was considered significant.

## 3. Results

### 3.1. Demographics and location of lesions 

Of the 209 patients in the ASA I–II risk groups, 41% were men and 59% were female with mean age of 50.57 ± 12.49 years, mean height of 168.27 ± 8.35 cm, and mean weight of 74.54 ± 11.1 kg. Of our patients, 99 were treated with L-Disq decompression at a single level while 110 procedures were performed at two levels. Single-level procedures were performed at L4–L5 level in 43 patients and L5–S1 level in 56 patients. On the other hand, two-level procedures were performed at L4–L5 + L5–S1 levels in 97 patients, L3–L4 + L4–L5 levels in 9 patients, and L3–L4 + L5–S1 levels in 4 patients. The demographic data are summarized in the Table. 

**Table:** 

	n (209)	
Mean age	50.57 ± 12.49	
Mean height (cm)	168.27 ± 8.35	
Mean weight (kg)	74.54 ± 11.16	
	n (209)	Percentage
Sex		
Male	88	42.1
Female	121	57.9
Level		
Single-level	99	47.36
L4–L5	43	20.57
L5–S1	56	26.8
Two-level	110	52.64
L4–L5 + L5–S1	97	46.4
L3–L4 + L4–L5	9	4.3
L3–L4 + L5–S1	4	1.9

None of the patients had previously undergone lumbar surgery at the spinal levels of the procedure.

All patients were also evaluated by PSS 12 months after the procedure, and 43 patients (20.6%) rated their satisfaction as very good, 126 patients (60.3%) as good, and 40 patients (19.1%) as moderate. The overall satisfaction rate was 80.9%. 

### 3.2. VAS score results

When compared to the initial values, VAS scores were found to be statistically significant for each treatment method at the 1st, 3rd, 6th, and 12th months (P = 0.001). When VAS scores were compared in binary between months, all differences were statistically significant (P = 0.001) aside from the difference between the 6th and 12th months (P = 0.394). A decrease in time-dependent VAS scores was detected although VAS scores increased between the 6th and 12th months after the procedure in some patients, but not to a significant level. 

### 3.3. ODI score results 

**Figure 1:**
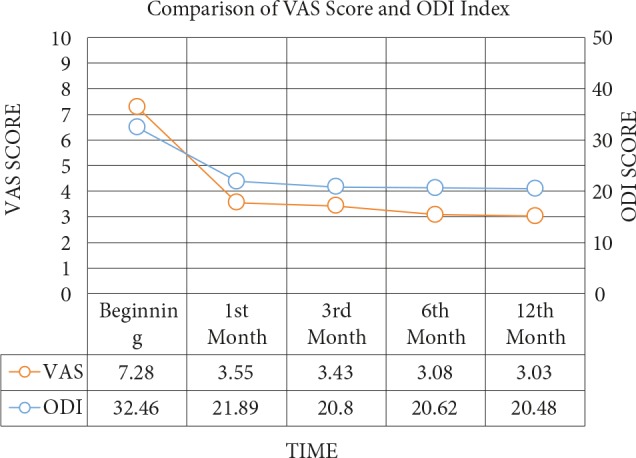
Comparison of visual analog scale (VAS) and Oswestry Disability Index (ODI) scores.

When compared to the initial values, ODI scores were found to be statistically significant for each treatment method at the 1st, 3rd, 6th, and 12th months (P = 0.001). When the ODI scores were compared in binary between months, the differences were statistically significant (P = 0.001), aside from the differences between the 3rd and 6th and the 6th and 12th months (P = 0.176 and P = 0.159, respectively). We concluded that the procedure had a significant effect in reducing ODI scores. Comparisons of VAS scores and ODI indexes are shown in Figure 1.

## 4. Discussion 

PDD methods are based on the principle that removal of the small amounts of discoid tissue will result in significant pain relief by reducing intradiscal pressure (5). Therefore, pressure on the nerve will be decreased and radicular findings will be reduced. There is evidence of clinically significant pain reduction, reduced disability of the patients, and safety of PDD (6–8).

In all procedures of our study, the therapeutic effect was significantly related to patient selection and the experience of the practitioner. The statistically significant reductions in VAS and ODI scores can be attributed to the long-term efficacy of the treatment with a follow-up of 12 months, as well as to the larger patient population in the present study when compared to previous studies. In the present study, the mean VAS scores reduced by 51.24%, 52.89%, 57.70%, and 58.38% at the 1st, 3rd, 6th, and 12th months, respectively, while the mean ODI score reduced from 32.46 points at the beginning to 20.48 points at the 12th month. 

A precise approach to the target region is crucial for a successful outcome and this may be achieved through the proper control of the navigable tip of the electrode. The electrode of L-Disq including the tip can be monitored under fluoroscopy (11). In our study, following the tip of the electrode by fluoroscopy made it easier to navigate towards the protruded disc. We believe that long-term continuity also contributed to the favorable results of our study. The target region for the insertion of the electrode was determined according to the current pathology of the herniated disc on MRI images. The interventional area is very close to the neural tissues at the posterior and vascular tissues at the anterior. Though the temperature rises significantly around the tip of the electrode, nerves or other structures outside the disc do not carry the risk of thermal damage if the tip remains intradiscal (13). We think that this will increase the success rate and safety of the procedure. 

The practitioner must be careful in order to prevent serious damage to neural and surrounding tissues. It is a great advantage that the tip of the electrode is visible during ablation and it should be prevented from moving out of the disc (11). Placing the tip of the electrode within the disc approximately 5–6 mm away from the border of the neural tissue provides an optimal benefit for safety (14).

**Figure 2:**
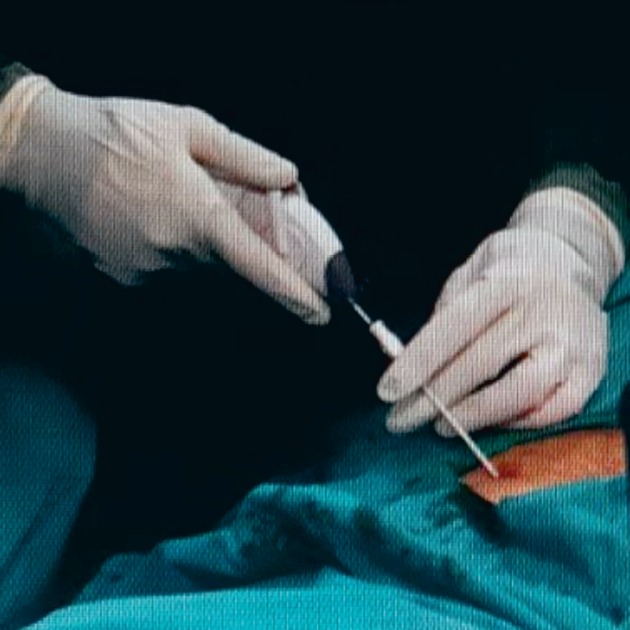
Application of L-Disq.

**Figure 3:**
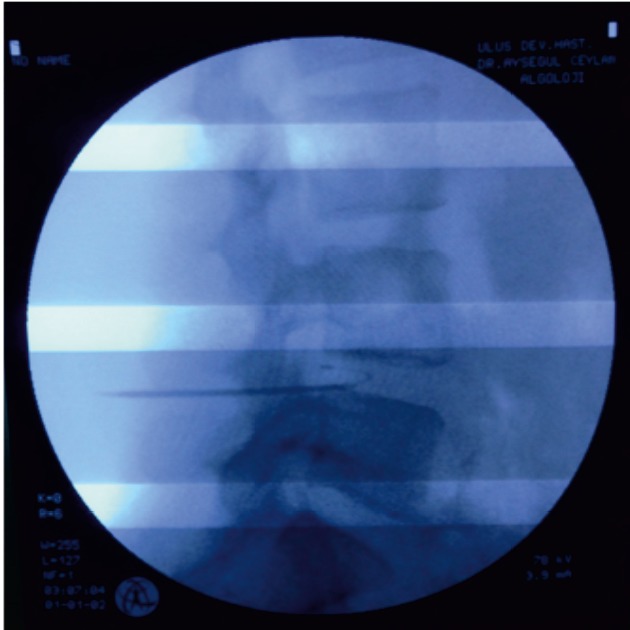
Image of L-Disq electrode under fluoroscopy.

Chen et al. (10) concluded that the volumetric removal of target discal tissue can be achieved without damage to the neighboring nuclei, rings, end plates, spinal cord, or nerve roots. The method of targeted ablation within the disc minimizes the amount of ablated discal tissue and fibrous damage to the outer ring, meaning that an adequate amount of nucleus pulposus is preserved and excessive reduction in discal height is prevented (5). Application of the L-Disq and an image of the L-Disq electrode under fluoroscopy are shown in Figures 2 and 3.

Lee et al. analyzed the data from 20 patients with discogenic waist pain who underwent ablation decompression using the L-Disq. When compared to the baseline values, the mean VAS score reduced from 7.55 to 3.60 points, and the mean ODI score reduced from 48.04 to 27.8 points. The authors concluded that significant pain relief and reductions in disability index could be achieved following ablation in patients with discogenic lower back pain (5). In addition to disc herniation detected by MRI, we also ablated the area of annular fissure in degenerated discs, if present. We suppose that treating the herniated disc together with the fissure in the same session may have increased our success rate.

In another study, Lee et al. reported their results of lumbar disc decompression using an L-Disq device in 25 patients with acute pain and extruded or protruded discs. The mean VAS score was 7.08 points at the beginning and reduced to 1.84 points at the 12th month, while the mean ODI score was 41.88 points at the beginning and reduced to 16.66 points at the 12th month (15). The results of our study were parallel to those of the two previous studies mentioned above.

Grönemeyer et al. reported the success rate of percutaneous laser discectomies (PLDDs) as 74% in 20 patients with HNP after a follow-up of 4 years (16). Although the results were similar to those of our present study, it has been shown that percutaneous laser nucleolysis offers an insufficient level of temperature control, which may lead to injury of adjacent tissues (17–19). This procedure comes with some disadvantages, such as moderate to severe intraoperative pain, thermal effects of the laser, postoperative back pain, spasm, and lack of visualization of the laser tip under fluoroscopy (20). 

Our patients experienced no muscular pain or burning during or after the procedure, and suffered no muscular spasms after the procedure. Accordingly, we believe that L-Disq is a more preferable technique in terms of patient comfort when compared to PLDD. The overall satisfaction rate from the L-Disq procedure was 80.9%. We also observed that our recommendations related to postprocedure lifestyle for the first 7–14 days helped our patients to reduce the spasms and inflammatory responses that may occur in this period, and no complications were seen in any of the patients discharged within 24 h after the procedure. 

Some authors in previous studies (21–23) did not observe a significant correlation between MRI findings and pain or various clinical signs. In our study, we did not perform MRI routinely for follow-up after the procedure. The pain and functional status was determined by clinical anamnesis and physical examination.

Although not statistically significant, our patients recorded increased VAS scores in their later follow-up appointments, but to evaluate this result more accurately, it would have been necessary to have noted the patient’s occupational status, social life, and living conditions, and to have followed their physical activities when recording the changes in VAS scores. This would have allowed us to classify the discal degeneration and healing rates more accurately, based on daily activities. The presence of such causal factors as vertebral instability, lifting heavy loads, and hard working conditions may lead to the recurrence or incomplete relief of discogenic pain. 

In conclusion, all of our patients benefited from the procedure. We believe that the most important factors playing a role in these promising results were performance of the procedure by the same experienced physician, giving weight to appropriate patient selection, the advantage of the access technique especially at the L5–S1 level, the navigable feature of the device, repairing the annular fissure together with the protruding disc, performing the procedure on a second level if detected on MRI, and performing regular follow-up after the procedure. We think that L-Disq is a safe and efficient procedure in patients with HNP when applied by an experienced practitioner because of its short duration, its own moving probe, the mechanical safety of the tip of the probe, and the opportunity to visualize the inside of the disc.
